# Electronic properties of single vacancy defect in boron nitride nanoribbons with edge perturbation

**DOI:** 10.1371/journal.pone.0305555

**Published:** 2024-08-09

**Authors:** Munawar Agus Riyadi, Yuki Wong, Sheng Xuan Khoo, Afiq Hamzah, Nurul Ezaila Alias, Cheng Siong Lim, Choon Min Cheong, Michael Loong Peng Tan

**Affiliations:** 1 Department of Electrical Engineering, Diponegoro University, Semarang, Indonesia; 2 Faculty of Electrical Engineering, Universiti Teknologi Malaysia, Skudai, Johor, Malaysia; 3 School of Engineering, INTI International College Penang, Penang, Malaysia; Federal University of ABC, BRAZIL

## Abstract

Two-dimensional material hexagonal boron nitride (h-BN), and its one-dimensional thin strips, boron nitride nanoribbons (BNNRs) are electrically insulating with high thermal stability, making them excellent thermal conductors suitable for high-temperature application. BNNRs are wide bandgap semiconductors with bandgaps ranging from 4 to 6 eV. This study investigates the electronic properties of BNNRs with single vacancy defects in armchair and zigzag configurations. The nearest-neighbour tight-binding model and numerical method were used to simulate the electronic properties of BNNRs with a single vacancy, including band structure and local density of states. The alpha and beta matrices were adjusted to account for missing boron or nitrogen atoms. Furthermore, a small perturbations were introduced to model the effects of impurities and edge imperfections. The simulation result from this work was compared with pristine BNNRs to examine the impact of a single vacancy on their electronic properties. The findings reveal that both armchair and zigzag BNNRs with single vacancy defects exhibit distorted band structures and local density of states due to the delocalization of *p_z_* orbitals. The valence bands show a higher concentration of nitrogen, while the conduction bands are richer in boron. These findings provide insights into how vacancy defects and edge perturbations can influence the electronic properties of BNNRs, which can guide the design and optimization of BNNR-based electronic devices in future research.

## 1. Introduction

Boron nitride (BN) is a typical III–V group compound with a 1:1 stoichiometry of boron and nitrogen [1, 2]. It has the highest thermal conductivity among electrical insulators [3, 4]. BN is similar to carbon in any lattice structure where it consists of a zero-dimensional cage, one-dimensional nanotube, two-dimensional monolayer and three-dimensional diamond-like crystal structure [1]. A h-BN sheet is analogous to graphene because it is isoelectronic and isomorphic to the graphene honeycomb lattice [5, 6]. h-BN is produced by hot pressing, with the material being slightly hygroscopic and machinable with conventional tooling [7]. By cutting a straight line, h-BN can be constructed into two types of BN nanoribbons (BNNRs), namely armchair BN nanoribbons (ABNNRs) and zigzag BN nanoribbons (ZBNNRs) [8–10]. BNNRs possess higher thermal stability, oxidation stability up to 800°C, chemical inertness [11] and excellent optical properties. In the BN structure, the boron and nitrogen atoms are connected with a strong covalent bond. However, the interlayers between the BN layers are held together with weak Van der Waals forces [12, 13]. Various defects can occur during the fabrication of nanoribbons, such as Stone-Wells defects, vacancies, linear defects, nanoholes or quantum antidots [14, 15]. Inevitably, these defects can influence the characteristics of the BNNRs, such as energy band structure, carrier mobility, and conductivity [16]. The electronic properties of pristine BNNRs were investigated, with edge perturbation introduced to modify these properties [17]. Edge perturbation involves creating disturbances on a regular structure to alter its motion, resulting in an imperfect structure on BNNRs [18]. This method effectively reduces the large energy bandgap of BN, enhancing its semiconductor characteristics [17].

The solutions of the Schrödinger equation, either using first-principles [10, 19] or semi-empirical methods, are the basis for most quantum transport models. For example, the popular non-equilibrium Green’s Function formalism [20] and the Usuki method. Solving the Schrödinger’s equation is a complicated task because it involves the solution of the integral for each energy state existing in the entire system. The mathematical analysis of the Schrödinger’s equation is more complicated than involving a small system if it is enlarged. A longer time and more effort are needed to study the Schrödinger equation, including the theory and the calculation parts. Many researchers are using the Schrödinger equation to investigate the band structure and electronic properties of BNNRs [21, 22]. In this study, the tight-binding method, particularly the Nearest-Neighbour Tight-Binding (NNTB) model, was used due to its lower computational cost compared to first-principles methods such as Density Functional Theory (DFT) [23]. When constructing the Hamiltonian equation, pristine BNNRs were modified to have single vacancy defect BNNRs. By intentionally introducing vacancies [24], we studied the effect on these imperfections and understood how they affect the behaviour of BNNRs. One of the nitrogen or boron atoms was removed from the BNNRs. The *t* term and energy on the site of boron and nitrogen in alpha and beta were modified accordingly when boron or nitrogen was removed. The *t* term became zero and the energy on the boron and nitrogen sites became infinite [25].

Significant distortions are revealed in the band structure and local density of states (DOS) attributed to the delocalization of *p_z_* orbitals through molecular dynamics simulation, exploring the impact of vacancies and edge perturbations on the electronic properties of BNNRs. Conversely, another study highlights the energetically favoured nature of nitrogen (N) vacancies over boron (B) vacancies and the induction of magnetic moments and spin polarization, particularly in non-edge vacancy configurations. This research investigates the structural, electronic, and magnetic properties of zigzag BN nanoribbons (ZBNNRs) with periodic vacancies using spin-polarized band structure and DOS calculations [26]. Their focus is on zigzag BN nanoribbons with periodic vacancies, employing first-principles projector-augmented wave (PAW) potentials within the density functional theory (DFT) framework under the generalized gradient approximation (GGA).

This paper is organized into three sections. Section 2 presents the mathematical model of the Hamiltonian matrix, band structure and density of states (DOS) of BNNRs with single vacancy using nearest-neighbour tight-binding (NNTB). Section 3 details all the simulation findings and Section 4 concludes with a summary of the findings.

## 2. Computational model

This section presents the computational modelling procedures and the mathematical equations used to obtain the band structures and DOS.

### 2.1 Tight-binding model

The NNTB model is a simplified method in solid-state physics that approximates the electronic structure of materials by considering only the interactions between an atom and its nearest neighbours in a crystal lattice [23]. The model was used to calculate the electronic properties of BNNRs because a short device length can be computed more efficiently. The model is based on the Schrödinger equation, shown in **Eq (1)** [21], which preserves the wave-like properties of electrons.

E{Ψ}=[H]{Ψ} (1)

The physical structure of the model is described by the Hamiltonian operator, H. This operator needs to be defined properly and paired with a suitable wave function, *Ψ* to obtain the energy spectrum [27].

### 2.2 Hamiltonian operator

A Hamiltonian matrix in **Eq (2)** can be obtained by defining the alpha and beta matrices.

$$H_{4-ABNNRs\;or\;ZBNNRs,L=3}=\left[\begin{array}{ccc} a & \beta & 0\\ \beta \prime & a & \beta \\ 0 & \beta \prime & a \end{array}\right]$$ (2)

**Fig 1:**
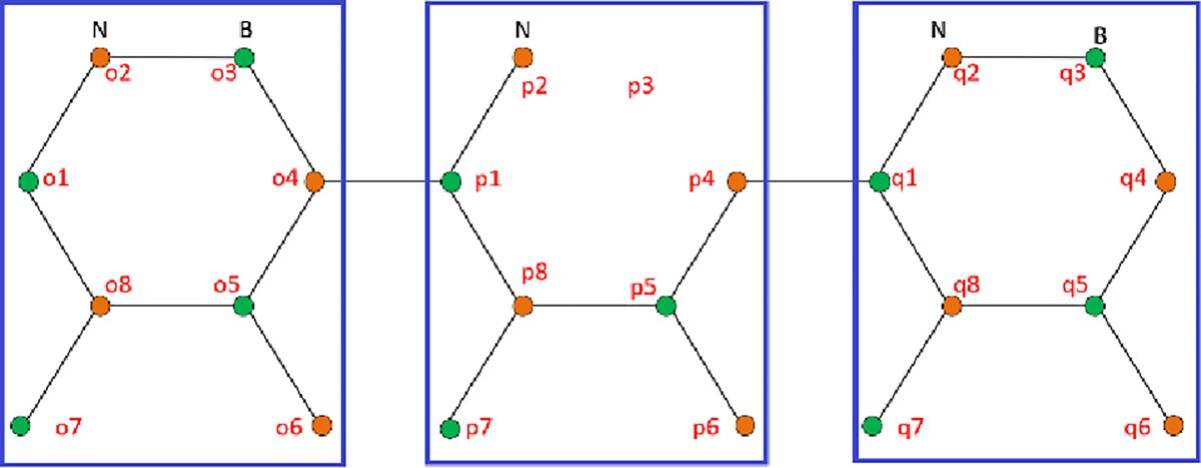
Schematic diagram of 4-ABNNRs with length = 3 and a boron atom vacancy at p3. The unit cells are highlighted and the atoms are numbered.

This approach enables the 2D BNNRs structure to be transformed into a 1D structure. This matrix contains α, *β* and *β*′ matrices, which represent the self-interacting unit cells, interactions between the unit cells and relationship to the other side of the alpha matrices, respectively. A clockwise sequence in numbering BN atoms was used to form the alpha matrix of monolayer BNNRs, as depicted in **Fig 1**.

The off-diagonals of the alpha matrix for the BNNRs were populted. For the 4-BNNRs and beyond, an additional term, *t*, was added to the alpha matrix because some of the π-orbitals have three nearest bonds within the defined boundary of the unit cell, as shown in **Eq (3)**. The self-interaction energy was set to infinity in the absence of the atom [25]. In **Eq (3)**, the p3 atom, a boron atom, is missing, leading to the replacement of boron’s self-interaction energy with infinity. Consequently, the term *t* is set to 0 due to the absence of bonds between the atoms.

$$\begin{array}{l} \begin{array}{llllllll} \;p1 & p2 & p3 & p4 & p5 & p6 & p7 & p8 \end{array}\\ \alpha_{4-ABNNRs}=\begin{array}{l} p1\\ p2\\ p3\\ p4\\ p5\\ p6\\ p7\\ p8 \end{array}[\begin{array}{cccccccc} \varepsilon_{B} & t & 0 & 0 & 0 & 0 & 0 & t\\ t & \varepsilon_{N} & 0 & 0 & 0 & 0 & 0 & 0\\ 0 & 0 & \infty & 0 & 0 & 0 & 0 & 0\\ 0 & 0 & 0 & \varepsilon_{N} & t & 0 & 0 & 0\\ 0 & 0 & 0 & t & \varepsilon_{B} & t & 0 & t\\ 0 & 0 & 0 & 0 & t & \varepsilon_{N} & t & 0\\ 0 & 0 & 0 & 0 & 0 & t & \varepsilon_{B} & t\\ t & 0 & 0 & 0 & t & 0 & t & \varepsilon_{N} \end{array}] \end{array}$$ (3)

**Fig 2:**
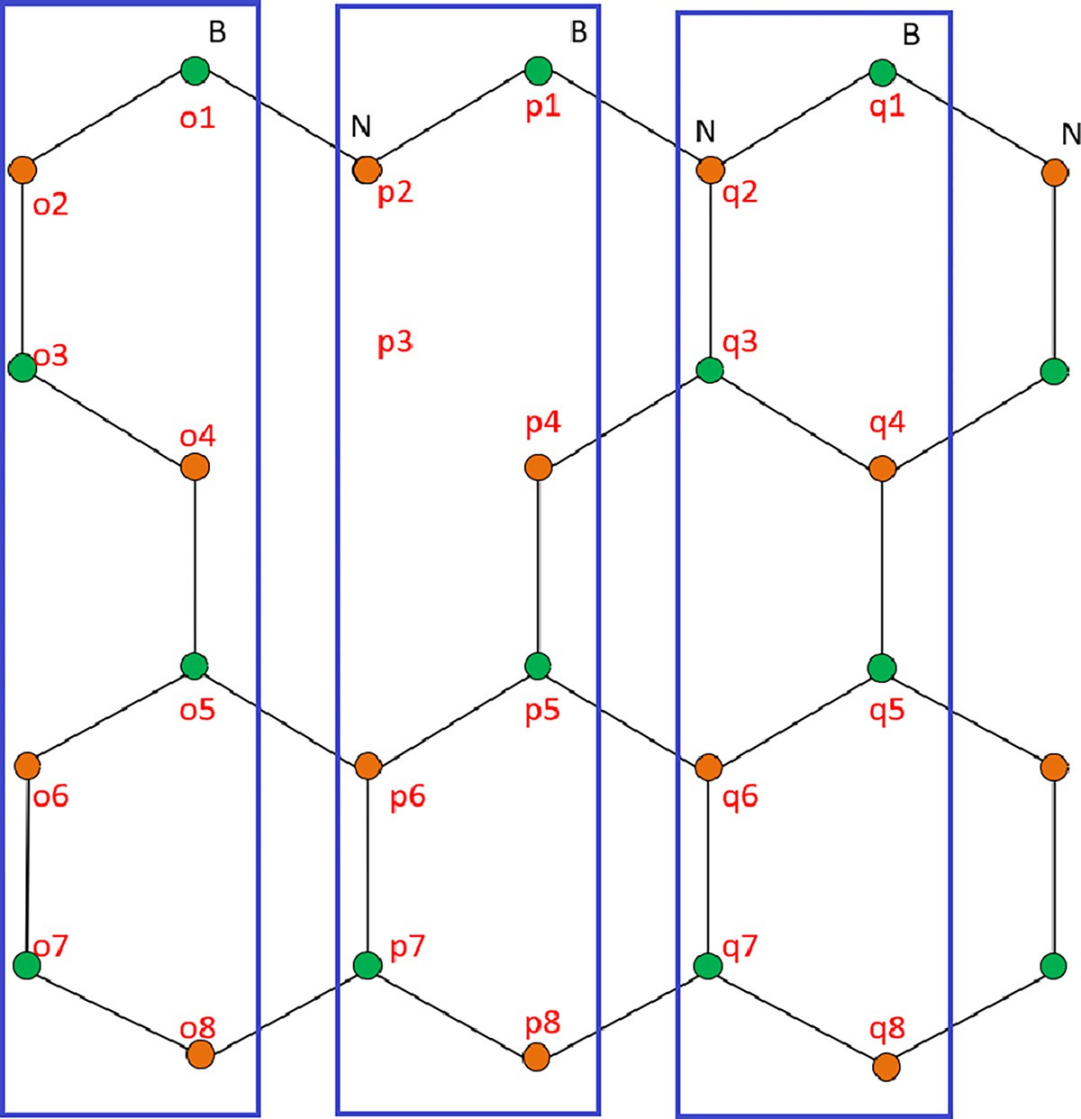
Schematic diagram of 4-ZBNNRs with length = 3 and a boron atom vacancy at p3. The unit cells are highlighted and the atoms are numbered.


The alpha matrix for the monolayer ZBNNRs is simple. **Fig 2** shows the construction of the α matrix for 4-ZBNNRs with boron vacancy at p3. The blue-highlighted boxes represent the ZBNNR unit cells. The structure of the alpha matrix for unit cell *p* is defined in **Eq (4)**, incorporating the self-interaction energies of both boron and nitrogen atoms.

$$\begin{array}{l} \;\begin{array}{llllllll} p1 & p2 & p3 & p4 & p5 & p6 & p7 & p8 \end{array}\\ \alpha_{4-ZBNNRs}=\begin{array}{l} p1\\ p2\\ p3\\ p4\\ p5\\ p6\\ p7\\ p8 \end{array}[\begin{array}{cccccccc} \varepsilon_{B} & t & 0 & 0 & 0 & 0 & 0 & 0\\ t & \varepsilon_{N} & 0 & 0 & 0 & 0 & 0 & 0\\ 0 & 0 & \infty & 0 & 0 & 0 & 0 & 0\\ 0 & 0 & 0 & \varepsilon_{N} & t & 0 & 0 & 0\\ 0 & 0 & 0 & t & \varepsilon_{B} & t & 0 & 0\\ 0 & 0 & 0 & 0 & t & \varepsilon_{N} & t & 0\\ 0 & 0 & 0 & 0 & 0 & t & \varepsilon_{B} & t\\ 0 & 0 & 0 & 0 & 0 & 0 & t & \varepsilon_{N} \end{array}] \end{array}$$ (4)

The beta matrix represents the interaction between the unit cells described by the alpha matrix. It accounts for the interaction sites for π-orbitals between the unit cells. To construct a beta matrix for ABNNR, the interactions between unit cell *o* and unit cell *p* are expressed in **Eq (5)**. The term in the matrix structure denotes the interaction between boron and nitrogen atoms from different unit cells. The relationship for the other side of the alpha matrix of ABNNR can be obtained by transposing the beta matrix in the **Eq (5)**. The beta matrix of ZBNNR is similar to that of ABNNR and is expressed in **Eq (6)**.


$$\begin{array}{l} \;\begin{array}{llllllll} q1 & q2 & q3 & q4 & q5 & q6 & q7 & q8 \end{array}\\ \beta_{4-ABNNRs}=\begin{array}{l} p1\\ p2\\ p3\\ p4\\ p5\\ p6\\ p7\\ p8 \end{array}[\begin{array}{cccccccc} 0 & 0 & 0 & 0 & 0 & 0 & 0 & 0\\ 0 & 0 & 0 & 0 & 0 & 0 & 0 & 0\\ 0 & 0 & 0 & 0 & 0 & 0 & 0 & 0\\ t & 0 & 0 & 0 & 0 & 0 & 0 & 0\\ 0 & 0 & 0 & 0 & 0 & 0 & 0 & 0\\ 0 & 0 & 0 & 0 & 0 & 0 & t & 0\\ 0 & 0 & 0 & 0 & 0 & 0 & 0 & 0\\ 0 & 0 & 0 & 0 & 0 & 0 & 0 & 0 \end{array}] \end{array}$$ (5)

$$\begin{array}{l} \;\begin{array}{llllllll} q1 & q2 & q3 & q4 & q5 & q6 & q7 & q8 \end{array}\\ \beta_{4-ZBNNRs}=\begin{array}{l} p1\\ p2\\ p3\\ p4\\ p5\\ p6\\ p7\\ p8 \end{array}[\begin{array}{cccccccc} 0 & t & 0 & 0 & 0 & 0 & 0 & 0\\ 0 & 0 & 0 & 0 & 0 & 0 & 0 & 0\\ 0 & 0 & 0 & 0 & 0 & 0 & 0 & 0\\ 0 & 0 & t & 0 & 0 & 0 & 0 & 0\\ 0 & 0 & 0 & 0 & 0 & t & 0 & 0\\ 0 & 0 & 0 & 0 & 0 & 0 & 0 & 0\\ 0 & 0 & 0 & 0 & 0 & 0 & 0 & 0\\ 0 & 0 & 0 & 0 & 0 & 0 & t & 0 \end{array}] \end{array}$$ (6)

The hopping integral between the neighbouring π-orbitals of BN atoms or between unit cells, *t*, is −2.45 eV. Moreover, the on-site energies for carbon and silicon are *E*_*B*_ = −6.64 *eV* and *E*_*N*_ = −11.47 *eV* [17], respectively, as presented in **Table 1.**

**Table 1:** Computed NNTB model parameters for monolayer BNNR.

*Parameters*	*t (eV)*	*E_B_ (eV)*	*E_N_ (eV)*
Semiconductor type	-2.45	-6.64	-11.47

### 2.3 Density of states

A localised DOS, *LDOS*(*E*), is the number of states occupied at a specific energy interval in a unit cell. The localised DOS can be simulated numerically using the delta function, δ (15) [28]. The *LDOS*(*E*) was calculated using **Eq (7)** here.

$$LDOS(E)=\sum_{i=1}^{N}\frac{1}{2\pi }\int \delta [E_{i}(k)-E]dk,$$ (7)

where the delta function, *δ*, is expressed as **Eq (8)**. Substitution of **Eq (8)** into **Eq (7)** leads to **Eq (9)**.


$$\delta [E_{i}(k)-E]=\frac{\eta_{G}}{{\left[E_{i}(k)-E\right]}^{2}+{\eta_{G}}^{2}}$$ (8)

$$LDOS(E)=\sum_{i=1}^{N}\frac{1}{2\pi }\sum_{all\;k}\frac{\eta_{G}}{{\left[E_{i}(k)-E\right]}^{2}+{\eta_{G}}^{2}}$$ (9)

## 3. Results and discussions

This section is divided into three parts. The band structures and local DOS of ABNNRs and ZBNNRs with vacancies are shown first, followed by edge perturbation applied to the selected atom.

### 3.1 Band structure and local DOS of BNNRs with a single vacancy

**Fig 3:**
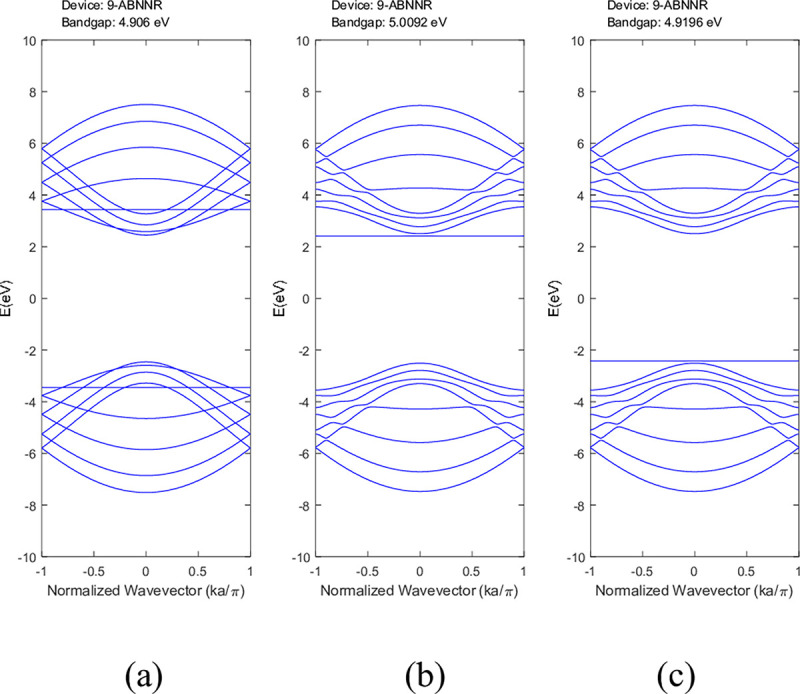
Band structure of 9-ABNNRs (a) pristine, with (b) a single nitrogen vacancy at p2 and (c) a single boron vacancy at p3.

The computational procedures described in Section 2 were used to simulate the band structures and DOS of BNNRs. The results were compared with the pristine BNNRs. **Fig 3** illustrated the band structure of 9-ABNNRs (a) pristine, with (b) a single nitrogen vacancy at p2 and (c) a single boron vacancy at p3. These bandgap values are consistent with the reported range of 4–6 eV for pristine BNNRs [29, 30].

**Fig 4:**
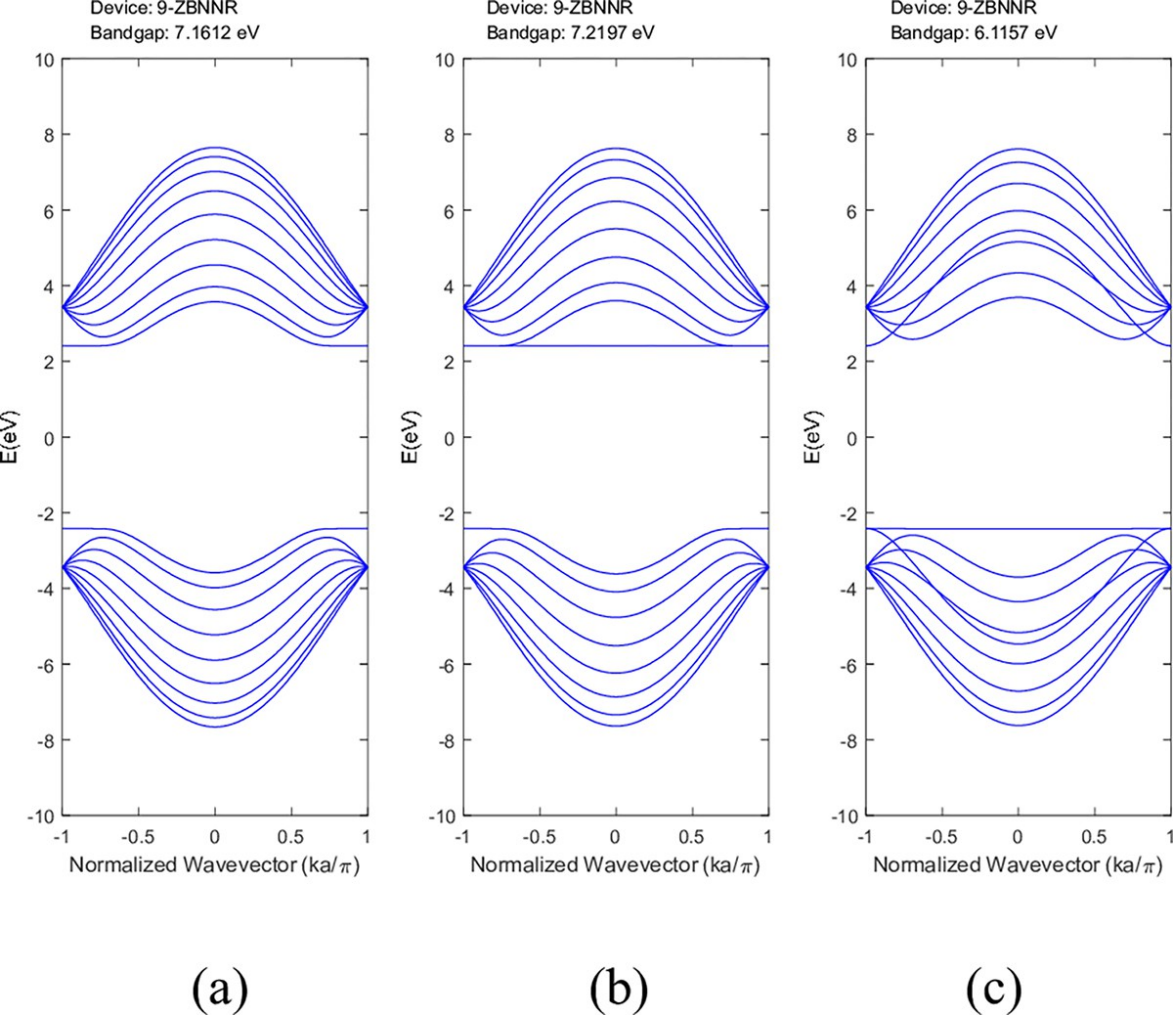
Band structure of 9-ZBNNRs (a) pristine, (b) with a single nitrogen vacancy at p2 and (c) with a single boron vacancy at p3.v.

The conduction and valence bands experienced distortion when either boron or nitrogen was missing in the ABNNRs. The first conduction band becomes flat when there is a nitrogen vacancy at location p2, while the first valence band flattens when there is a boron vacancy at location p3. **Fig 4** depicts the band structure of 9-ZBNNRs (a) pristine, (b) with a single nitrogen vacancy at p2 and (c) with a single boron vacancy at p3. Similar to **Fig 3**, the conduction and valence bands in **Fig 4** were also altered when either boron or nitrogen was absent in the ZBNNRs. The first conduction band becomes a horizontal line with a nitrogen vacancy at location p2, and the first valence band exhibits the same behaviour with a boron vacancy at location p3, as seen in the provided band structure plots.

**Fig 5:**
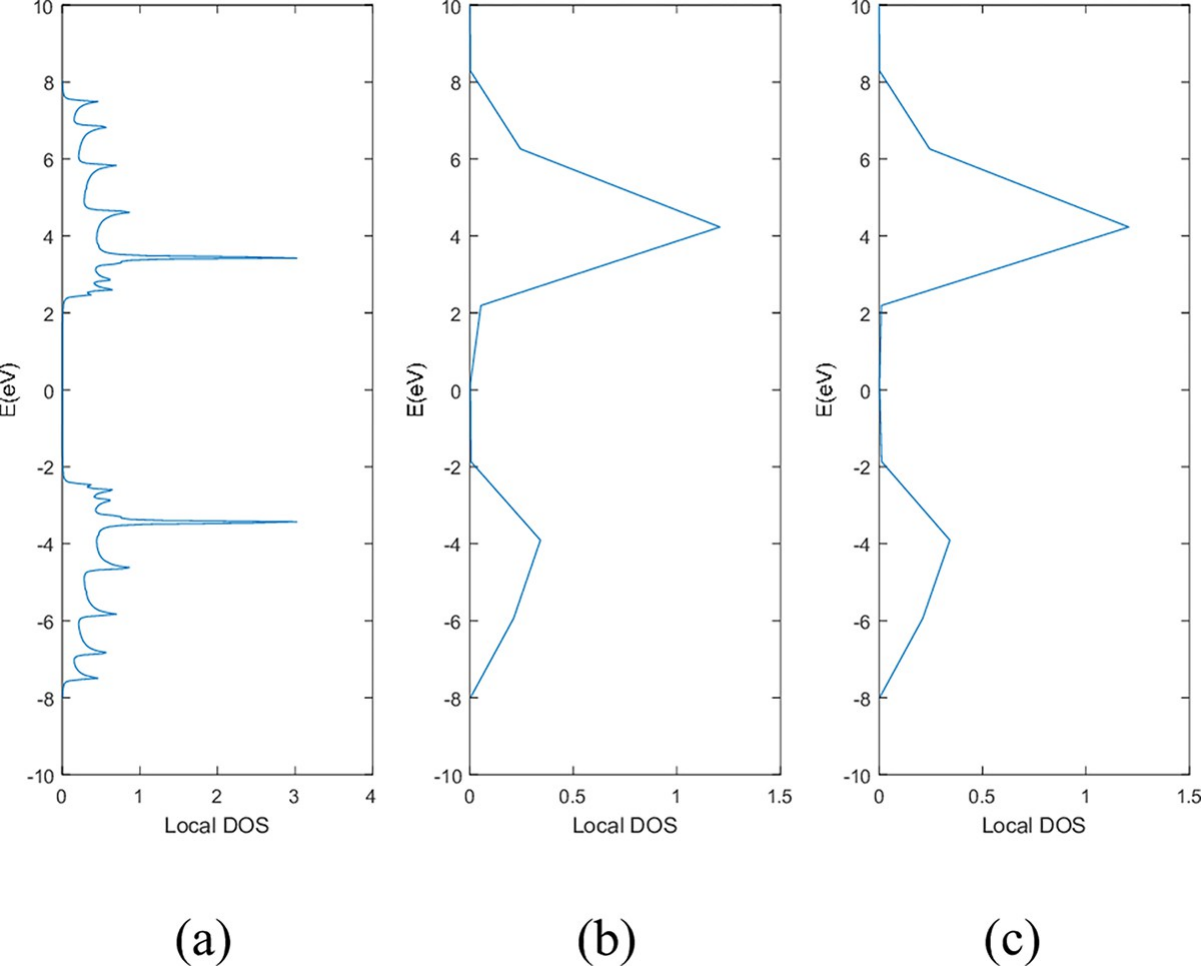
Local DOS of 9-ABNNRs (a) pristine, (b) with a single nitrogen vacancy at p2 and (c) with a single boron vacancy at p3.

**Fig 6:**
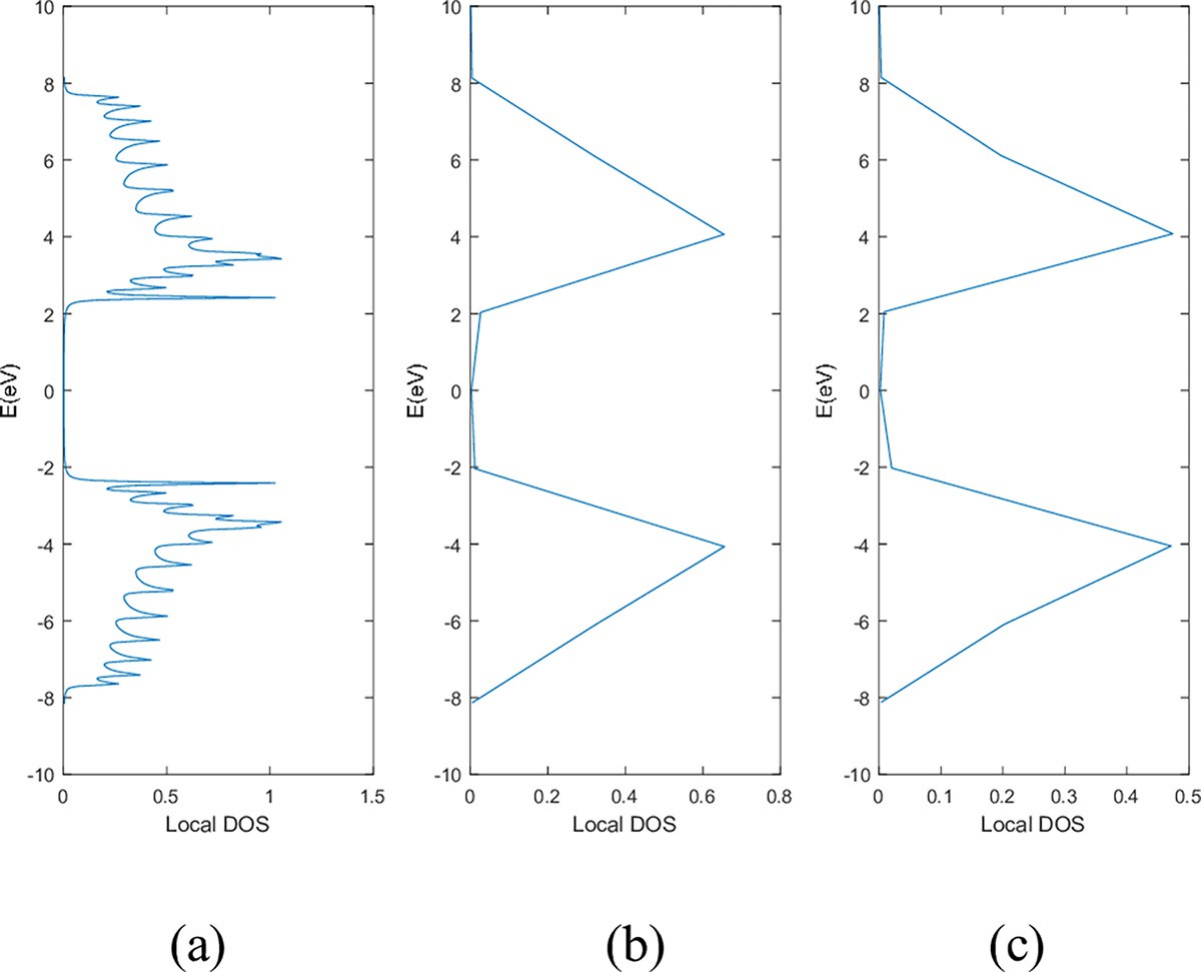
Local DOS of 9-ZBNNRs (a) pristine, (b) with a single nitrogen vacancy at p2 and (c) with single boron vacancy at p3.

**Figs 5** and **6** show the local DOS of (a) pristine, (b) with a single nitrogen vacancy at p2 and (c) with a single boron vacancy at p3 for 9-ABNNRs and 9-ZBNNRs respectively. The conduction and valence bands in **Fig 5** show distortions when there is a boron and nitrogen atom vacancy in 9-ABNNRs. The continuous curves shown in pristine 9-ABNNRs are no longer observed when there is a boron or nitrogen vacancy in 9-ABNNRs. This result is similar to 9-ZBNNRs, as shown in **Fig 6**. In pristine nanoribbons, abrupt spikes in the density of states, known as van Hove singularities, exist. However, in non-pristine nanoribbons, these singularities are broadened and diminished. This is due to a sudden increase or decrease in electronic states near specific energy levels caused by the crystal lattice’s geometry and band structure.

### 3.2 Band structure and local DOS of BNNRs with a single vacancy and edge perturbation

**Fig 7:**
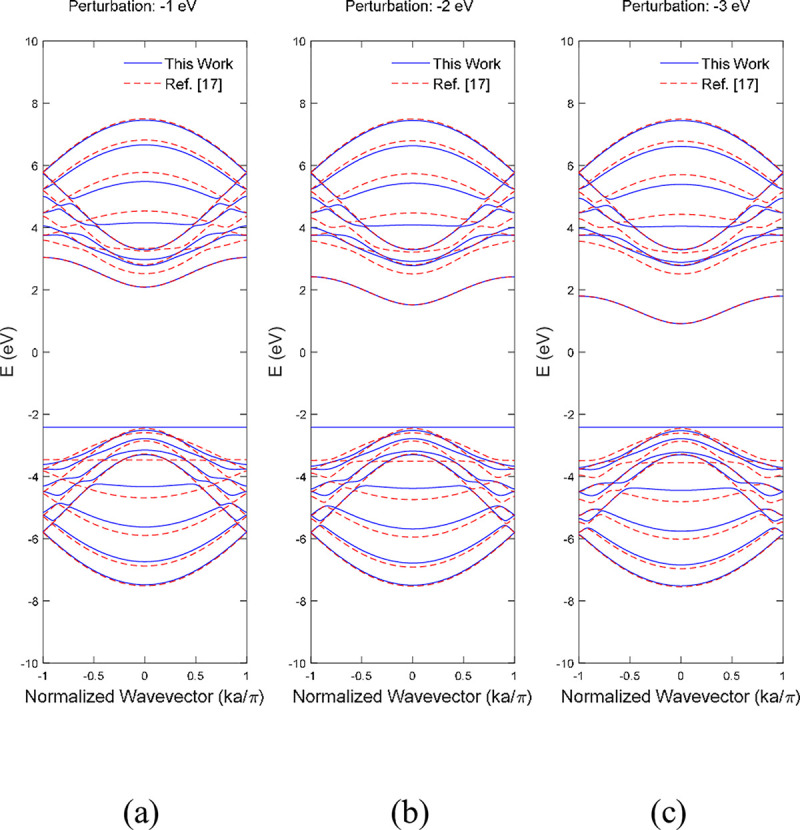
Band structure after edge perturbation of (a) −1 eV, (b) −2 eV and (c) −3 eV on the bottom edge boron atoms of single boron vacancy 9-ABNNRs at p3. The red dotted lines represent the benchmark from Ref. [17] without vacancy.

**Fig 8:**
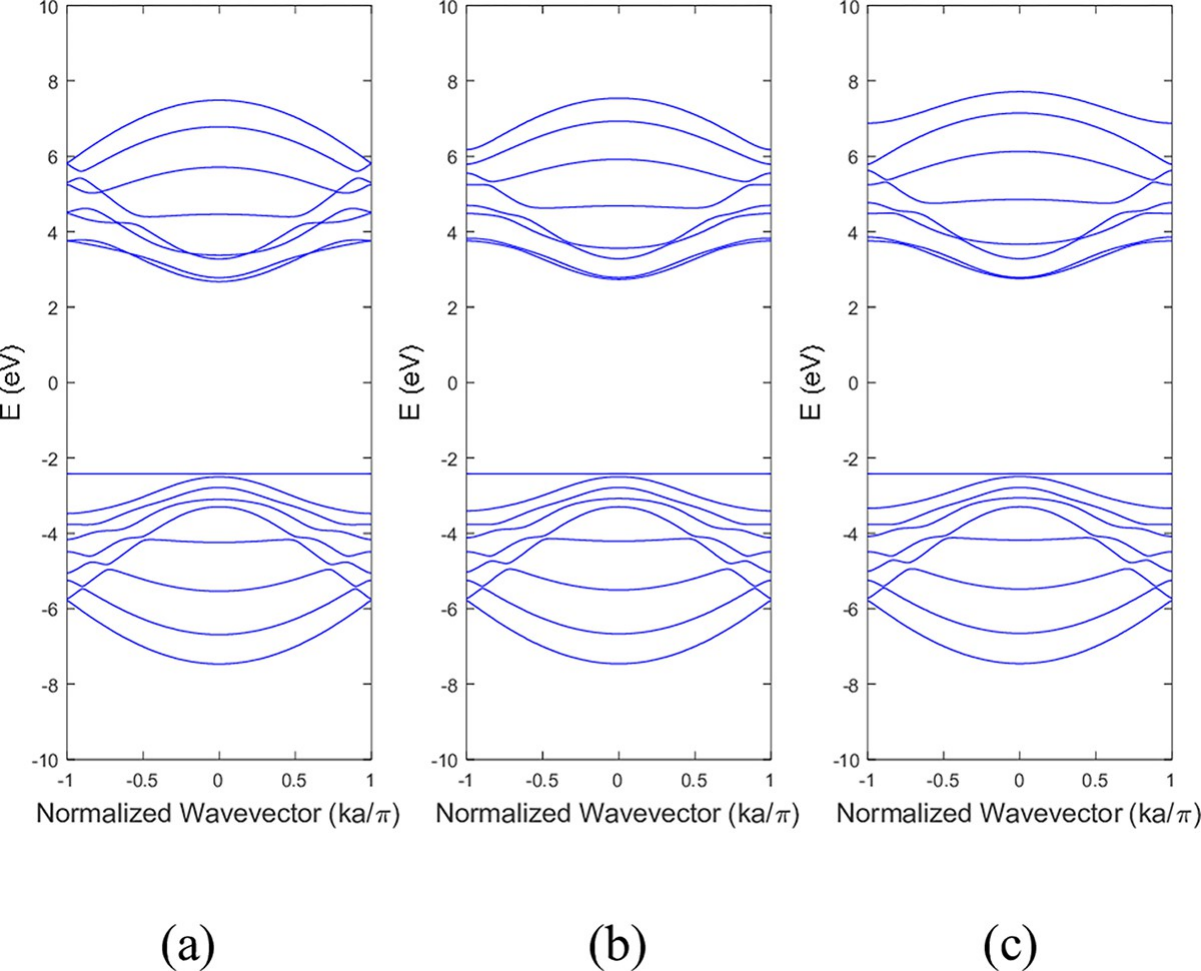
Band structure after the edge perturbation of (a) +1 eV, (b) +2 eV and (c) +3 eV on the bottom edge boron atoms of a single boron vacancy 9-ABNNRs at p3.

A small perturbation effect within a range of ±3 eV was applied at the on the bottom edge boron atoms of single boron vacancy 9-ABNNRs at p3 respectively in **Figs 7** and **8**. For single boron and nitrogen vacancy BNNRs, the on-site energies of the atoms at the edges were adjusted to reflect the impact of impurities and imperfections. This adjustment modifies the alpha matrix in the tight-binding model, where the self-interaction energies of the perturbed atoms are altered. This method effectively simulates the impacts of impurities and imperfections on the edges of BNNRs.

The band structure depicted in **Fig 7** shows the first conduction band was pulled down when the strength of perturbation increased from −1 eV to −3 eV. Previously published work [17] showed similar behaviour where the first conduction band shown in dotted red lines was pulled to the Fermi energy level when the perturbation strength was increased from −1 eV to −3 eV. The valence band remained stable when the strength increased from −1 eV to −3 eV.

The valence band remained unchanged as the edge perturbation strength increased from +1 eV to +3 eV as illustrated in **Fig 8**. On the other hand, the conduction band experienced a lesser degree of distortion when the strength increased from +1 eV to +3 eV.

**Fig 9:**
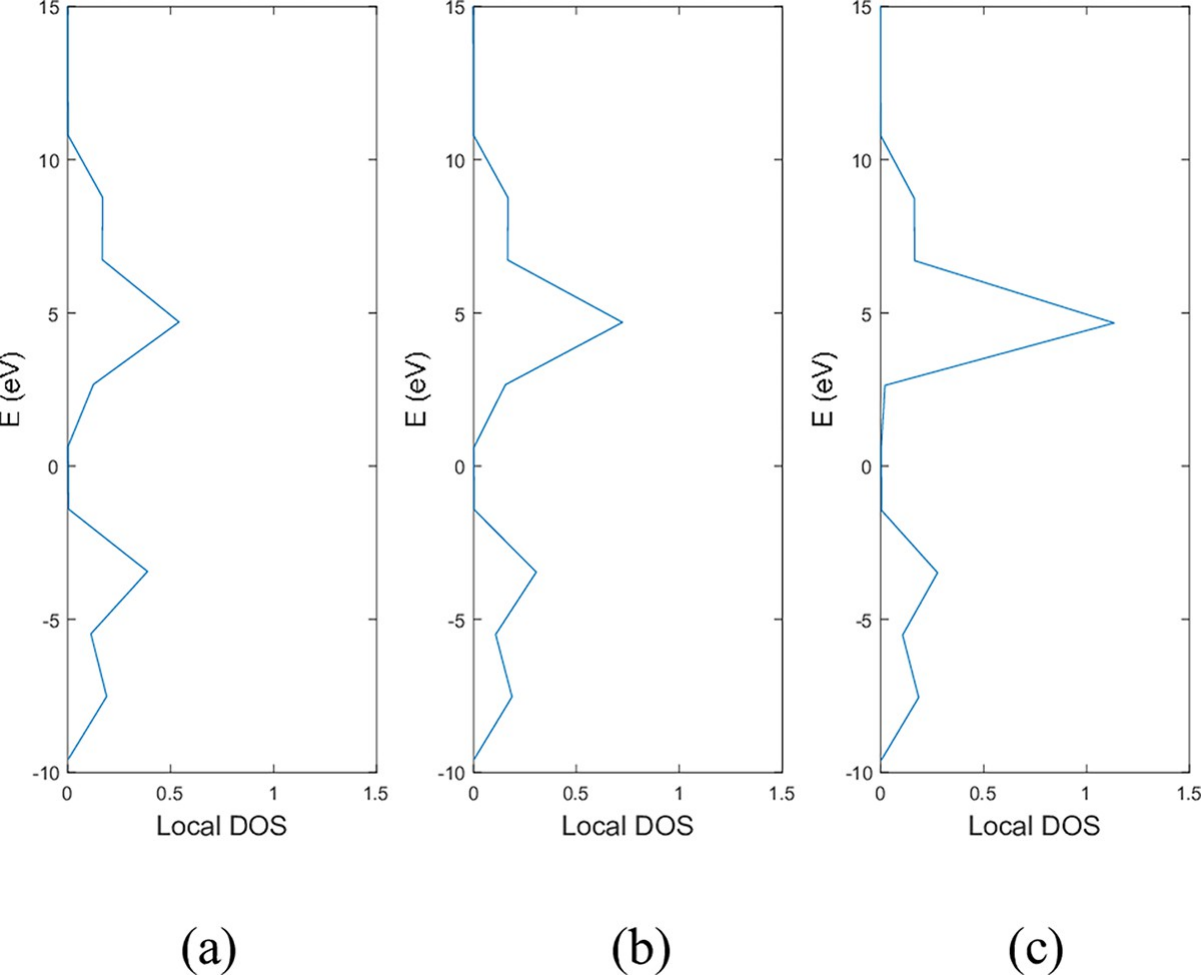
Local DOS after edge perturbation of (a) −1 eV, (b) −2 eV and (c) −3 eV on bottom edge boron atoms of a single boron vacancy 9-ABNNRs at p3.

**Fig 10:**
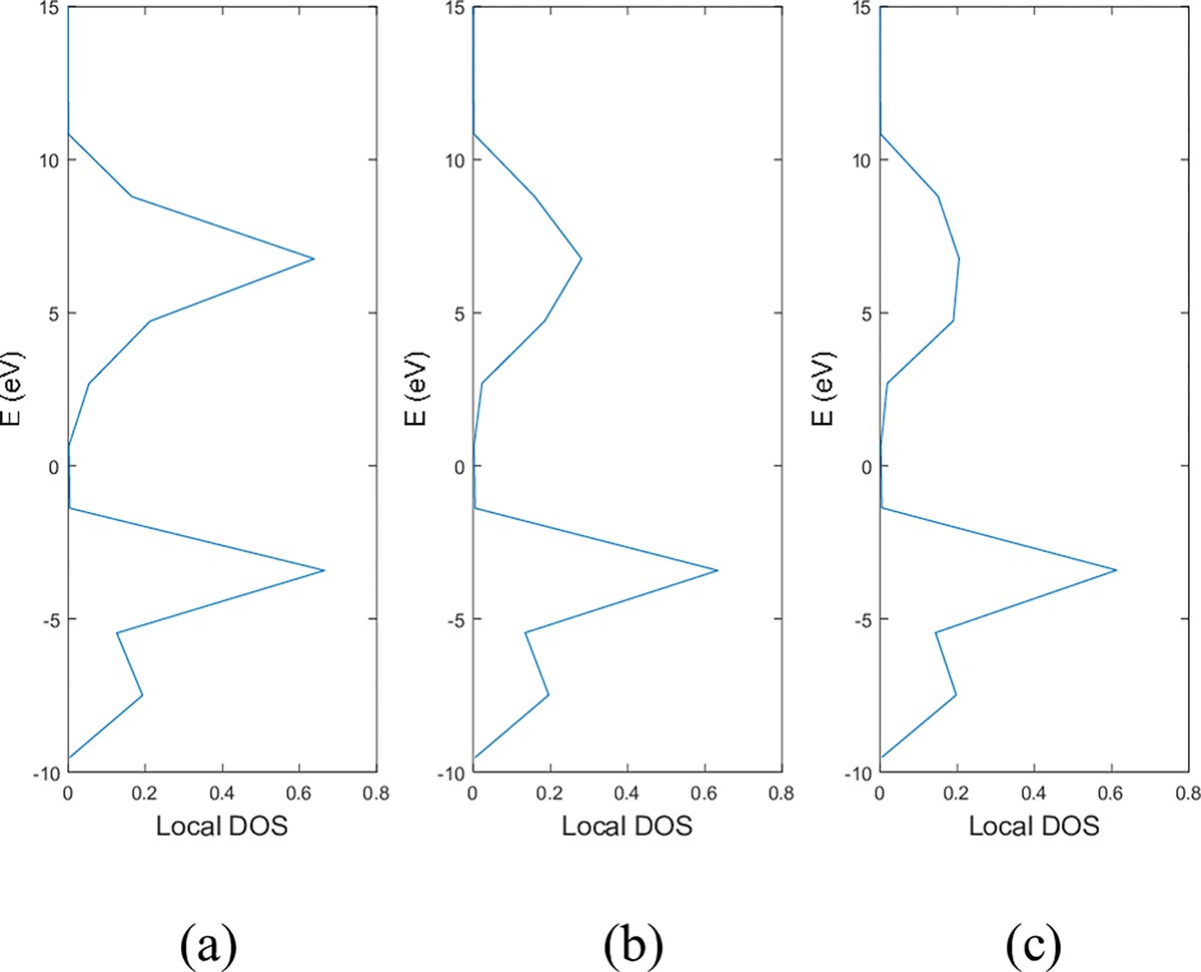
Local DOS after the edge perturbation of (a) +1 eV, (b) +2 eV and (c) +3 eV on the bottom edge boron atoms of a single boron vacancy 9-ABNNRs at p3.

**Figs 9** and **10** shows the local DOS of 9-ABNNRs with the impact of edge perturbation effect within a range of ±3 eV on bottom edge boron atoms of a single boron vacancy at p3, respectively. The peak value of the conduction band in **Fig 9** increased when the perturbation strength increased from −1 eV to −3 eV. The valence band did not show any large differences in both **Figs 9** and **10**. However, the peak value of the conduction band decreased when the perturbation strength increased from +1 eV to +3 eV as shown in **Fig 10**.

**Fig 11:**
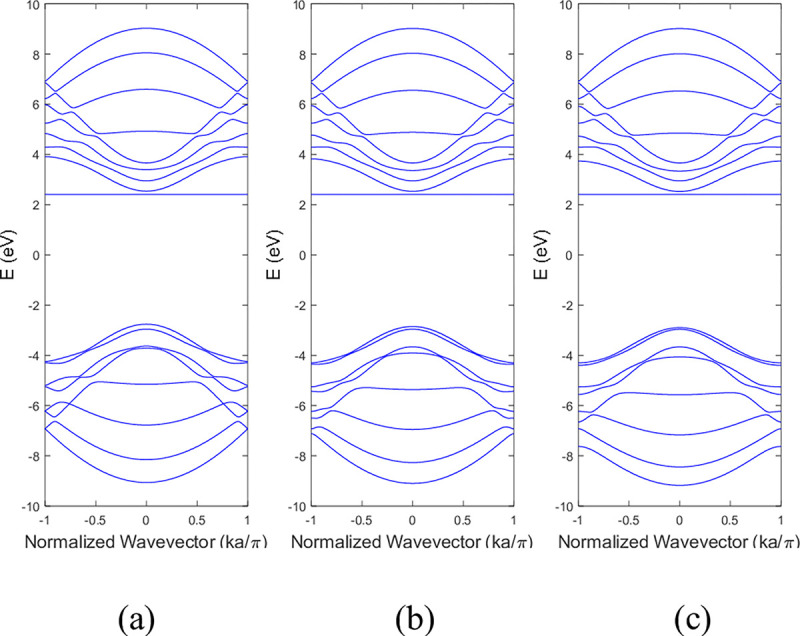
Band structure after the edge perturbation of (a) −1 eV, (b) −2 eV and (c) −3 eV on the bottom edge nitrogen atoms of a single nitrogen vacancy 9-ABNNRs at p2.

**Fig 12:**
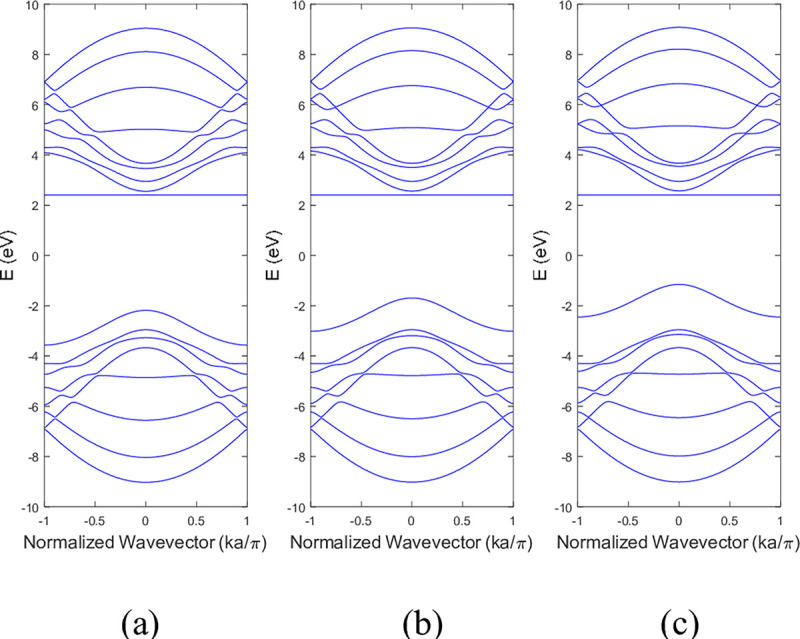
Band structure after the edge perturbation of (a) +1 eV, (b) +2 eV and (c) +3 eV on the bottom edge nitrogen atoms of a single nitrogen vacancy 9-ABNNRs at p2.

**Figs 11** and **12** shows the band structure of 9-ABNNRs with the impact of edge perturbation effect within a range of ±3 eV on bottom edge nitrogen atoms of a single nitrogen vacancy at p2, respectively. As shown in **Fig 11**, the conduction band remained unchanged when the strength of the edge perturbation was increased from −1 eV to −3 eV. The valence band experienced less distortion when the strength was increased from −1 eV to −3 eV.

**Fig 12** was shifted closer to the Fermi energy level when the perturbation strength was increased from +1 eV to +3 eV. However, there was no significant change in the conduction band when the strength was increased from +1 eV to +3 eV.

**Fig 13:**
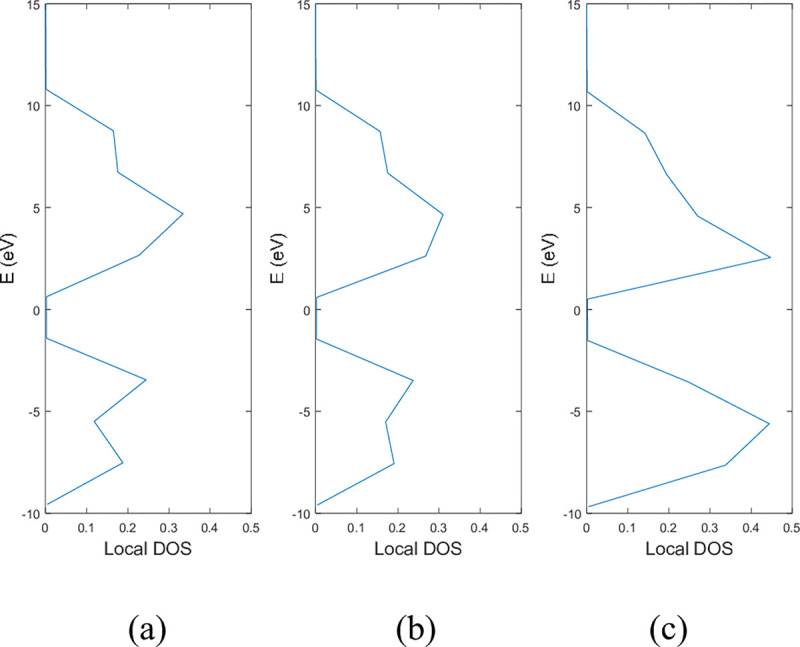
Local DOS after the edge perturbation of (a) −1 eV, (b) −2 eV and (c) −3 eV on the bottom edge nitrogen atoms of a single nitrogen vacancy 9-ABNNRs at p2.

**Fig 14:**
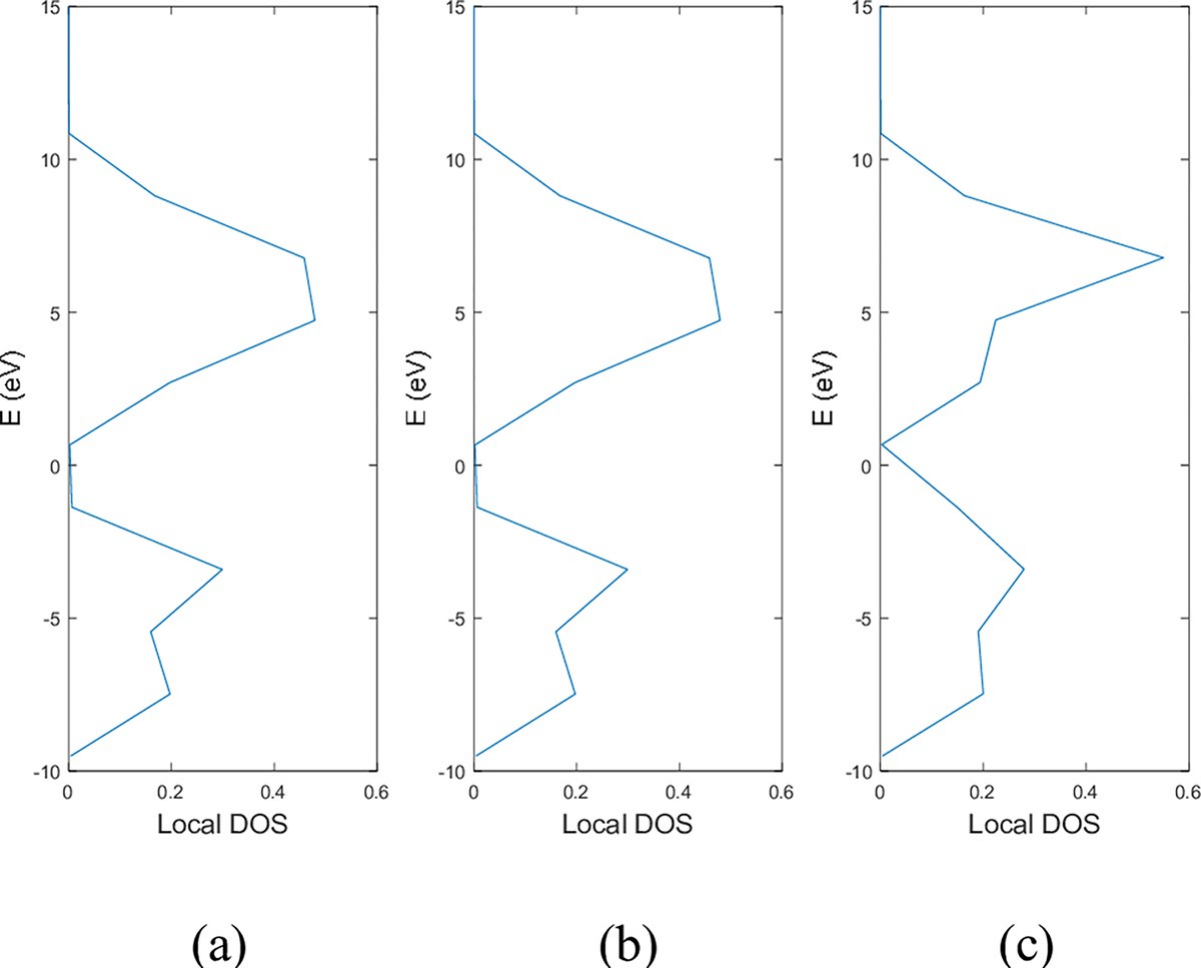
Local DOS after the edge perturbation of (a) +1 eV, (b) +2 eV and (c) +3 eV on the bottom edge nitrogen atoms of a single nitrogen vacancy 9-ABNNRs at p2.

**Figs 13** and **14** shows the band structure of 9-ABNNRs with the impact of edge perturbation effect within a range of ±3 eV on bottom edge nitrogen atoms of a single nitrogen vacancy at p2, respectively. The conduction and valence bands deconvolute a peak when the perturbation strength was at −3 eV, as shown in **Fig 13**.

The peak value of the conduction band in **Fig 14** was slightly higher at a perturbation strength of +3 eV compared to the peak value at a perturbation strength of +1 eV and +2 eV. The large spike in the local DOS at higher perturbation suggests that this state and its surrounding states are boron-rich due to the missing nitrogen atom.

**Fig 15:**
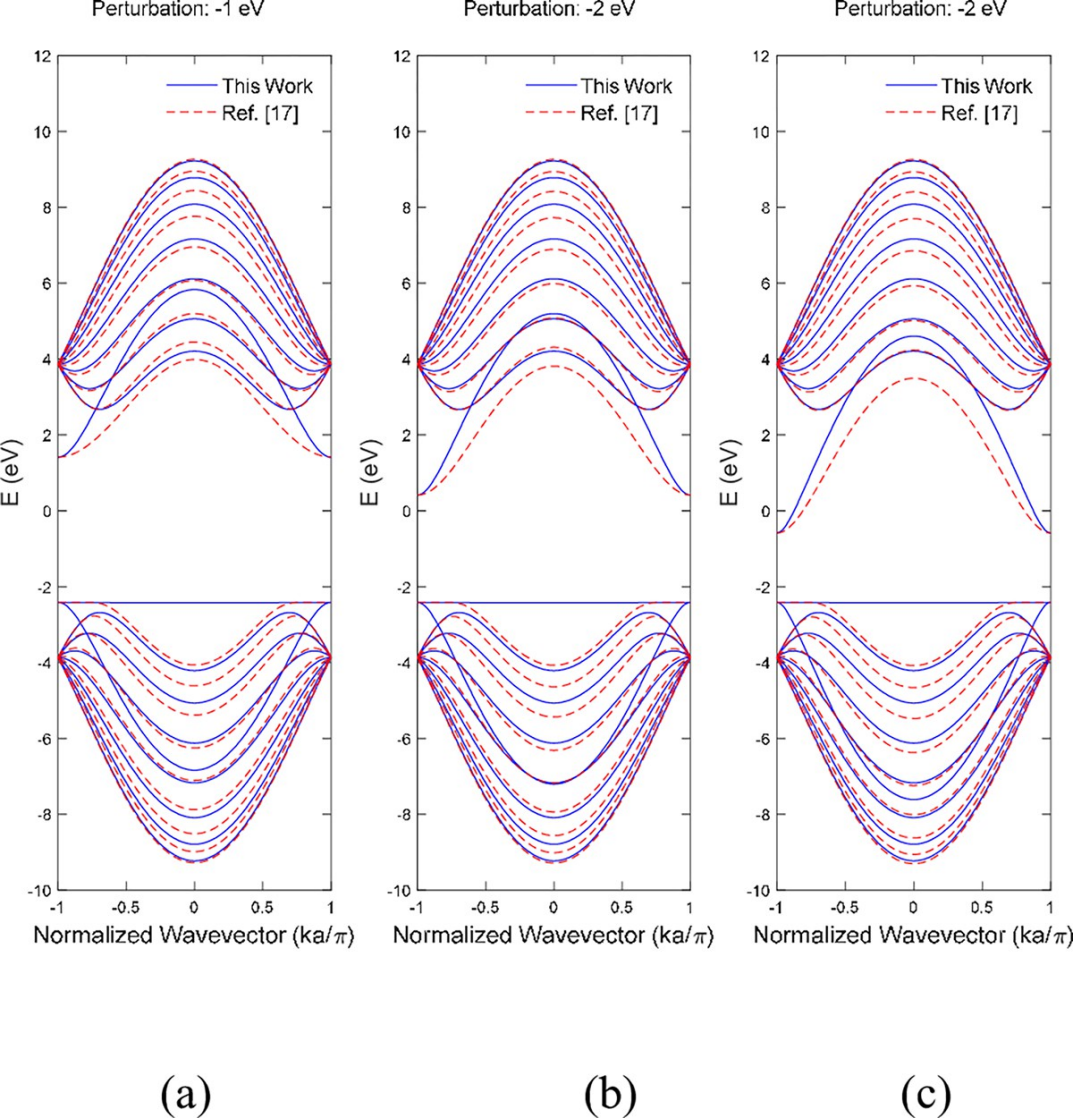
Band structure after edge perturbation of (a) −1 eV, (b) −2 eV and (c) −3 eV on the top edge boron atoms of a single boron vacancy 9-ZBNNRs at p3. The red dotted lines represent the benchmark from Ref. [17] without a vacancy.

**Fig 16:**
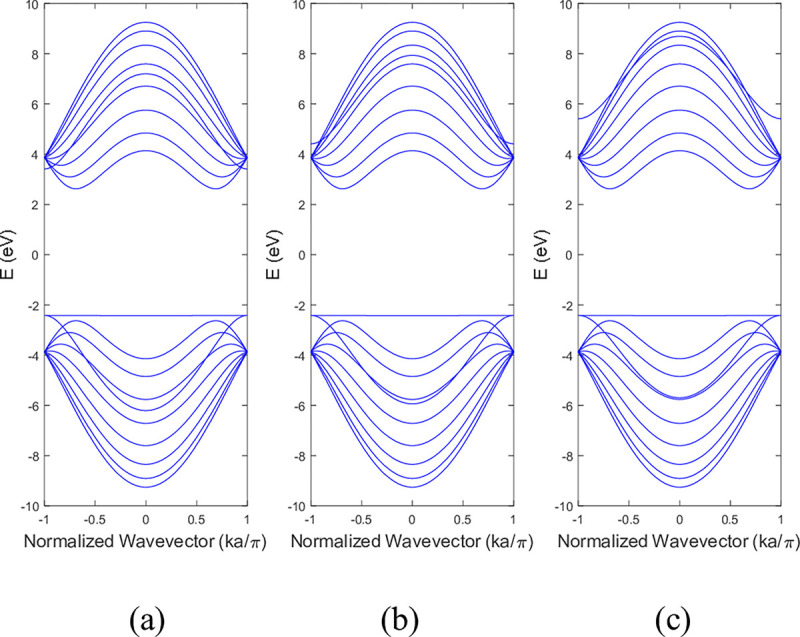
Band structure after the edge perturbation of (a) +1 eV and (b) +2 eV, (c) +3 eV on the top edge boron atoms of a single boron vacancy 9-ZBNNRs at p3.

**Figs 15** and **16** depict the band structure of 9-ZBNNRs affected by the edge perturbation within a range of ±3 eV on the top edge boron atoms of a single boron vacancy at p3, respectively. The band structure depicted in **Fig 15** shows that increasing the perturbation strength from -1 eV to -3 eV caused the first conduction band to decrease and approach the Fermi energy level. This is consistent with previous findings as seen in the reference work (8). The valence band remained stable when the strength was increased from −1 eV to −3 eV.

**Fig 16** reveals that increasing the edge perturbation strength from +1 eV to +3 eV had no effect on the valence band, however, the conduction band showed less deviation at the higher strength of +3 eV.

**Fig 17:**
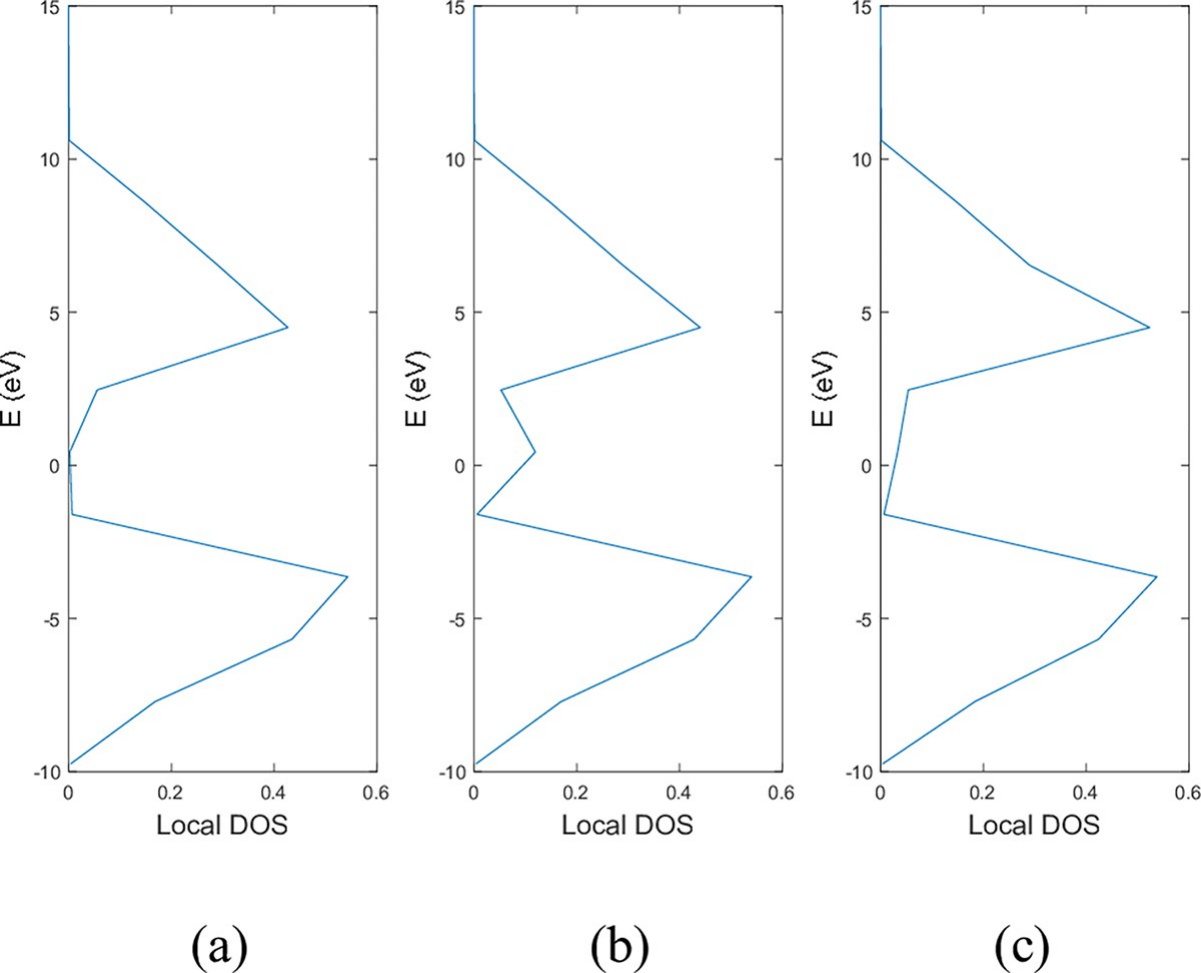
Local DOS after the edge perturbation of (a) −1 eV, (b) −2 eV and (c) −3 eV on the top edge boron atoms of a single boron vacancy 9-ZBNNRs at p3.

**Fig 18:**
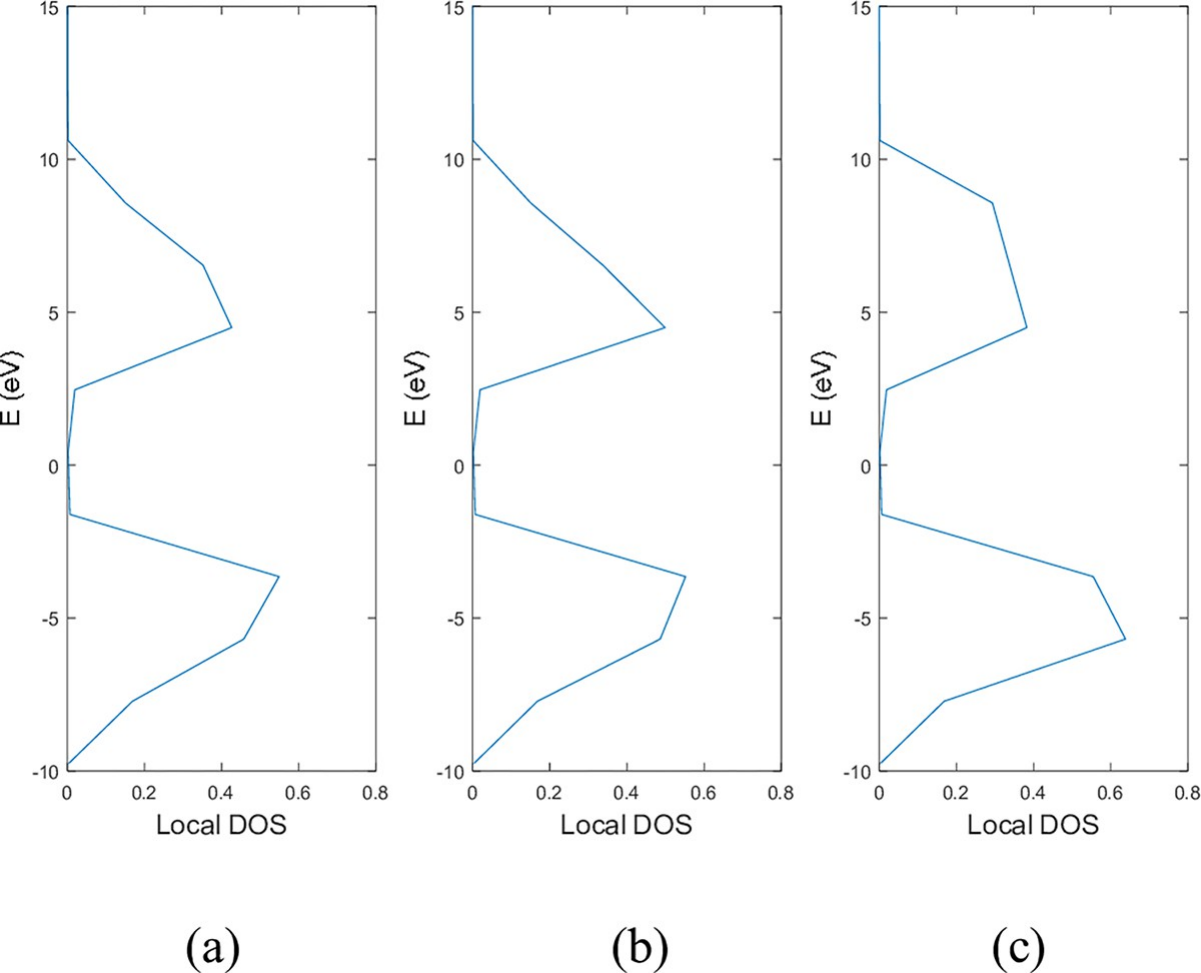
Local DOS after the edge perturbation of (a) +1 eV, (b) +2 eV and (c) +3 eV on the top edge boron atoms of a single boron vacancy 9-ZBNNRs at p3.

**Figs 17** and **18** depict the local DOS of 9-ZBNNRs with the impact of edge perturbation within a range of ±3 eV on the top edge boron atoms of a single boron vacancy at p3, respectively. As seen in **Fig 17,** increasing the perturbation strength from −1 eV to −3 eV did not produce a noticeable change in the conduction and valence bands.

On the other hand**, Fig 18** shows that increasing the perturbation strength from +1 eV to +3 eV resulted in a slight increase in the peak values of the conduction and valence bands. The large spike in the local DOS particularly in the valence band, in this case, indicates that there is a higher concentration of nitrogen atoms due to the missing boron atom.

**Fig 19:**
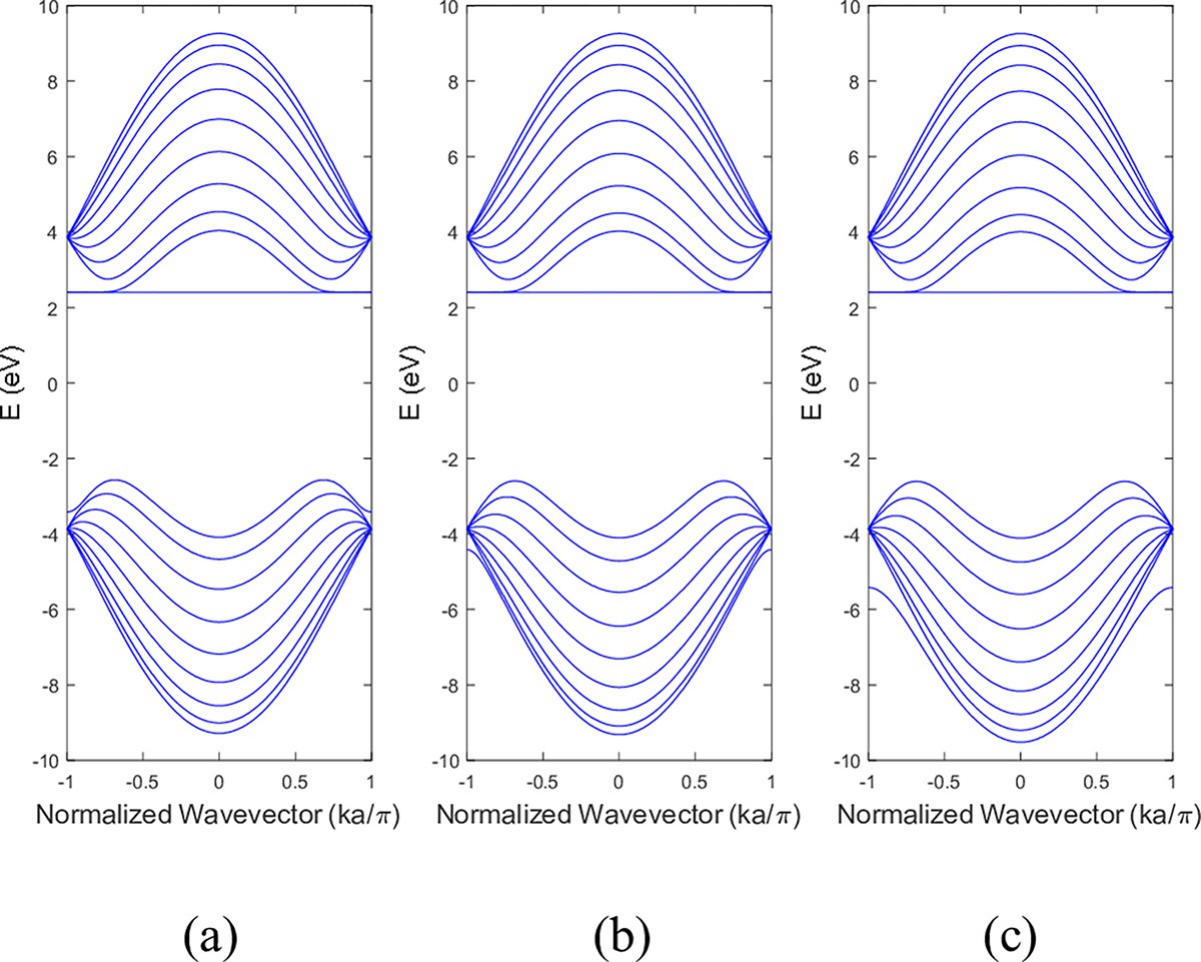
Band structure after the edge perturbation of (a) −1 eV, (b) −2 eV, (c) −3 eV on the bottom edge nitrogen atoms of a single nitrogen vacancy 9-ZBNNRs at p2.

**Fig 20:**
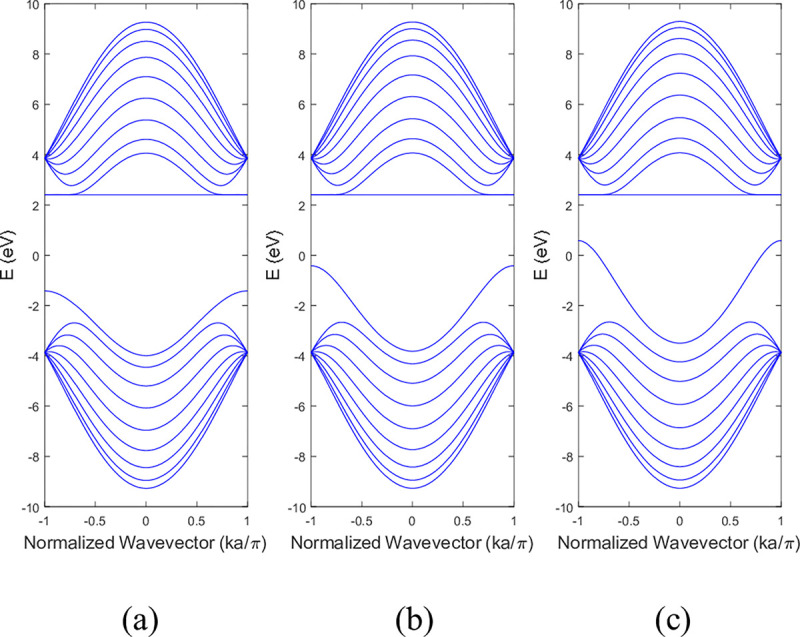
Band structure after the edge perturbation of (a) +1 eV, (b) +2 eV, (c) +3 eV on the bottom edge nitrogen atoms of a single nitrogen vacancy 9-ZBNNRs at p2.

**Figs 19** and **20** depict the band structure of 9-ZBNNRs with the impact of edge perturbation within a range of ±3 eV on the bottom edge nitrogen atoms of a single nitrogen vacancy at p2, respectively. In **Fig 19**, the conduction band did not change and the valence band showed less distortion when the perturbation strength was increased from −1 eV to −3 eV.

In **Fig 20**, the first valence band was pulled near the Fermi energy level when the perturbation strength was increased from +1 eV to +3 eV. However, the conduction band did not change significantly when the strength was increased from +1 eV to +3 eV.

**Fig 21:**
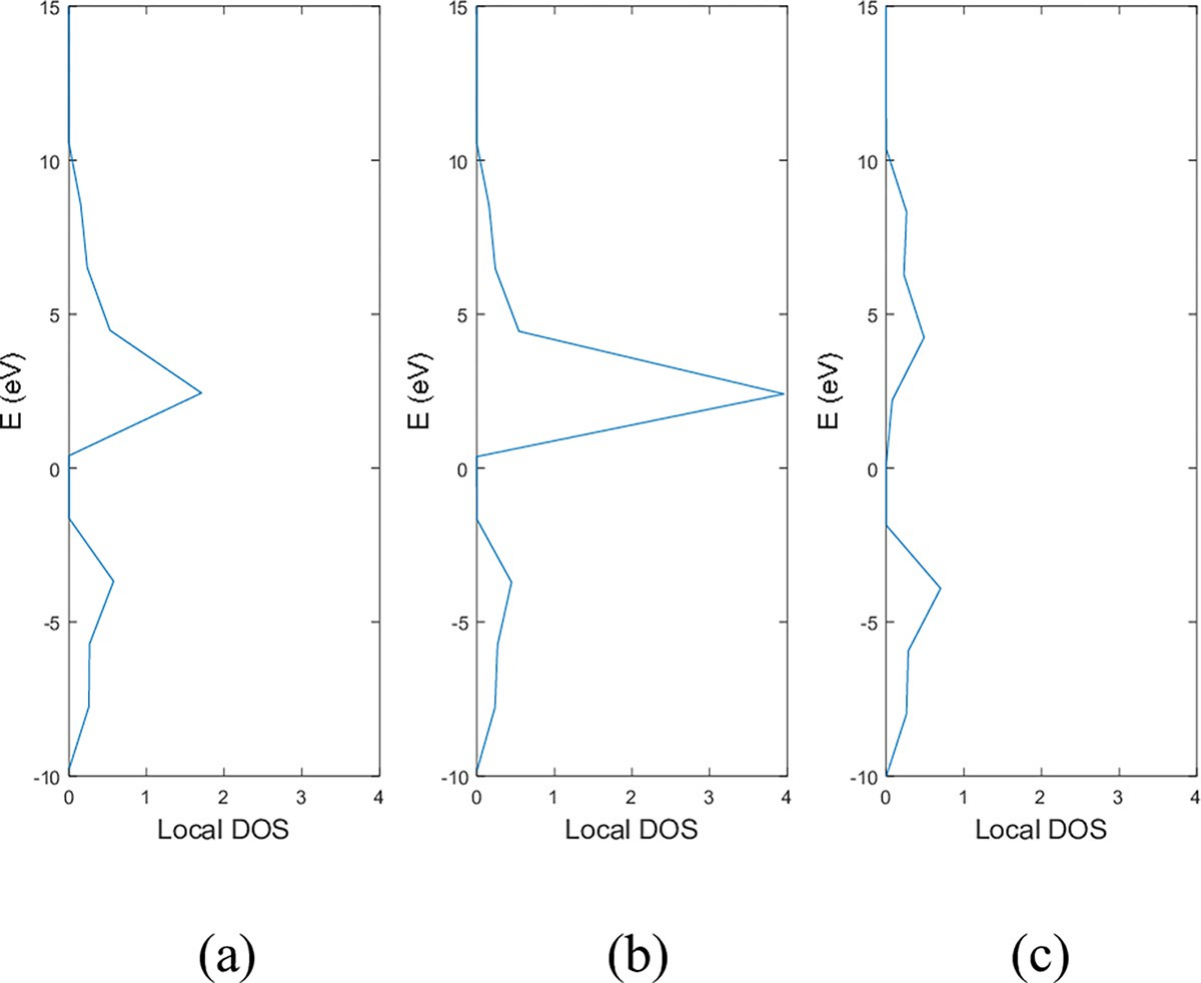
Local DOS after the edge perturbation of (a) −1 eV, (b) −2 eV, (c) −3 eV on the bottom edge nitrogen atoms of a single nitrogen vacancy 9-ZBNNRs at p2.

**Fig 22:**
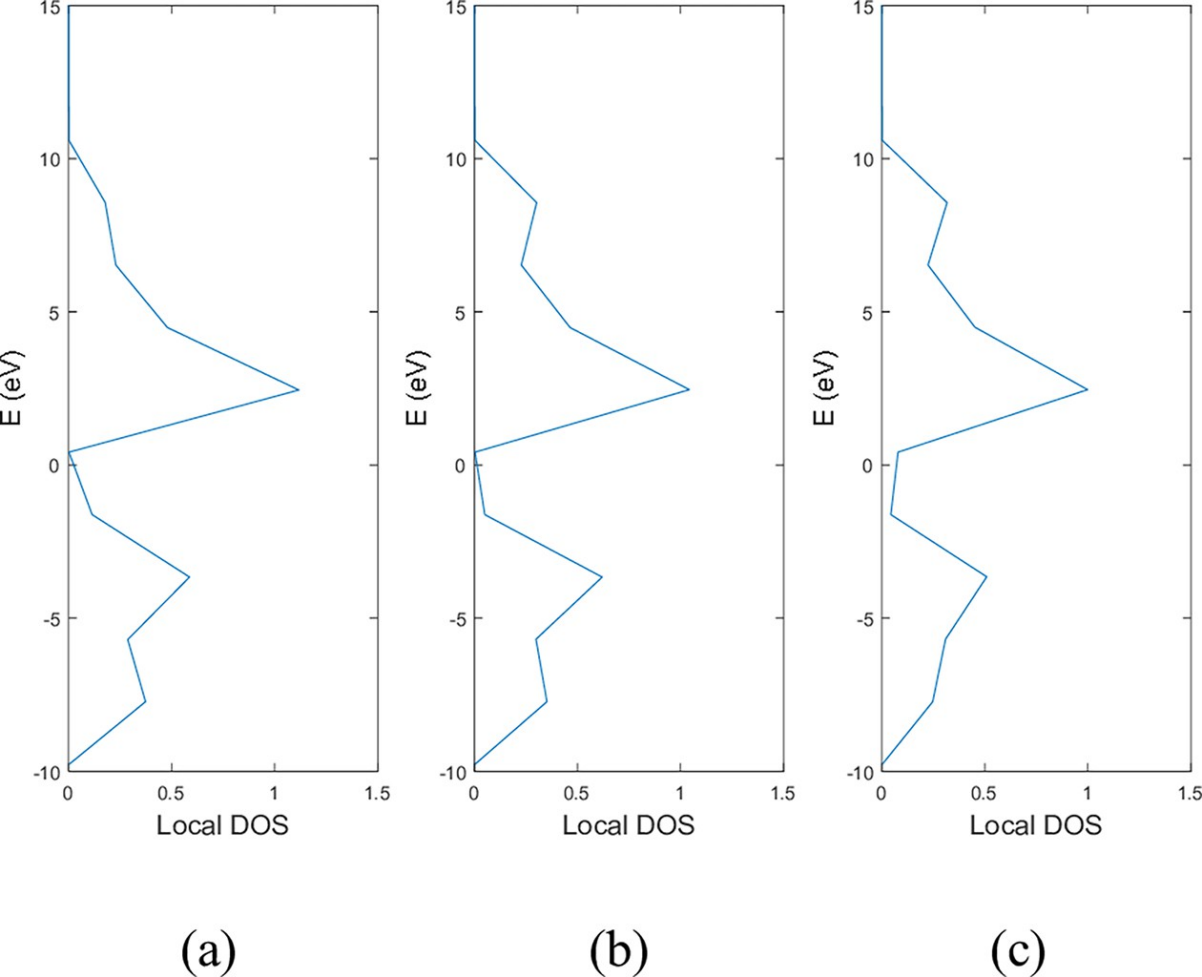
Local DOS after the edge perturbation of (a) +1 eV, (b) +2 eV, (c) +3 eV on the bottom edge nitrogen atoms of a single nitrogen vacancy 9-ZBNNRs at p2.

**Figs 21** and **22** depict the local DOS of 9-ZBNNRs affected by the edge perturbation within a range of ±3 eV on the bottom edge nitrogen atoms of a single nitrogen vacancy at p2, respectively. The peak value of the conduction band in **Fig 21** increased when the strength was increased from −1 eV to −2 eV, but the peak value of the conduction band in **Fig 21(C)** decreased when the perturbation strength reached −3 eV. The absence of an edge atom along with a high level of perturbation had a significant impact on the transport characteristics of the system, which was different from the effect of missing inner atom in **Fig 17**.

**Fig 22** demonstrates that the conduction and valence bands remained stable when the perturbation strength was increased from +1 eV to +3 eV.

## 4. Conclusion

The electronic properties of BNNRs with a single vacancy for armchair and zigzag-edged orientations were obtained using the numerical NNTB model. These properties include band structures and local DOS. For ABNNRs and ZBNNRs with a single vacancy, the band structure was distorted in the conduction band or valence band. For ABNNRs and ZBNNRs with a single vacancy at p2 or p3 and applied edge perturbation, the conduction band from the band structure was pulled toward or away from the Fermi level, depending on the applied perturbation strength. The perturbation causes the delocalization of *p_z_* orbitals on the band structure and density of states (DOS). As the *p_z_* orbital energies approach the Fermi level, they can become distorted and the band structure and DOS can be affected. The presence of a single vacancy also altered the distribution of atoms, where the valence bands have a higher concentration of nitrogen and the conduction bands have a higher concentration of boron. This led to changes in the electronic properties of BNNRs that are observed in these findings and provide insight into the behaviour of BNNRs and their potential applications in electronics and other fields.
